# How perceived risk influences college students' preventive behavior: Novel data of COVID-19 campus lockdown from Wuhan, China

**DOI:** 10.3389/fpubh.2023.1029049

**Published:** 2023-03-13

**Authors:** Lanxing Zhang, Xiaoyu Cheng, Zhuangzhuang Li

**Affiliations:** ^1^School of Physical Education, Central China Normal University, Wuhan, China; ^2^School of Physical Education, Wuhan Sports University, Wuhan, China

**Keywords:** perceived risk, preventive behavior, negative affect, positive affect, physical exercise

## Abstract

Following preventive behaviors is a key measure to protect people from infectious diseases. Protection motivation theory (PMT) suggests that perceived risk motivates individuals to take protective measures. The COVID-19 pandemic has posed unprecedented stress to the public, and changes in perceived risk may be more pronounced among college students than among other groups due to the related campus lockdown. With 1,119 college students recruited as research subjects, a quantitative research was conducted in Wuhan, China, to deduce the relationship between the perceived risk and preventive behavior of college students, as well as between the mediation effect of individual affect and the moderating effect of physical exercise. The results showed that the preventive behavior of college students was significantly affected by perceived risk, and both positive affect and negative affect played a mediating role between perceived risk and preventive behavior. Specifically, positive affect aided the relationship between perceived risk and preventive behavior, negative affect was detrimental to their relationship, and the mediation effect of positive affect is significantly higher than that of negative affect. Furthermore, physical exercise played a moderating role in the mediation effects of positive affect and negative affect. Therefore, appropriate measures should be taken to strengthen Chinese college students' perceived risk and provide them with corresponding guidance. The importance of physical exercise should also be emphasized to help college students with low perceived risk reduce negative affect, increase positive affect, and promote their preventive behavior.

## 1. Introduction

In 2022, the pandemic situation became more pressing because of its continuing mutation, shorter incubation period, and higher rates of asymptomatic infection. With the beginning of a new semester, some colleges and universities in China had to adopt a 1- to 3-month campus lockdown policy in response to the local outbreak of the COVID-19 pandemic, and college students had to take some actions to cope with this situation accordingly. The importance of preventive behavior [such as wearing masks when going out (1), actively disinfecting (2), and maintaining proper social distancing (3)] has become a global means to protect personal health and control infectious diseases. In general, college students are not recognized as a high-risk group for the COVID-19 pandemic. According to researchers from the United States, Bangladesh, and Poland, college students experience great stress in terms of mental (4, 5) and social adjustment (6), which can affect their daily behavior (7, 8). Nazione et al. (9) found the effective impact of information exposure on preventive behavior (PB); however, how the mental factors affect the PB of COVID-19 remains to be explored. The persistent recurrence of the COVID-19 pandemic provides a realistic social scenario for the study.

According to the protection motivation theory (PMT), perceived risk (PR) is a crucial factor in promoting an individual's PB (10, 11). PR refers to people's concerns or worries about an event (12) and is also regarded as an individual's subjective expectation of potential losses (13). In the event of a major public health emergency, those who perceive themselves to be at high risk for infectious diseases are more likely to take proactive actions such as maintaining personal hygiene (14), wearing masks (15), and vaccinating (16); that is, the higher the PR of an individual is, the more positive their PB will be during the COVID-19 pandemic. An online U.S. survey confirmed this action, and people even put aside their political inclinations and followed official recommendations when they perceived themselves to be at a higher risk of contracting COVID-19 (17). Further research found that women (18), middle-aged individuals, and older individuals (19) showed higher levels of PR and PB. Moreover, the impact of the pandemic on individual mental health and behavioral performance is long-lasting, and the relationship between the level of PR and individual PB also changes at different stages of public health events (14, 20). A longitudinal study (21) showed that PR motivated PB in the early stages of the COVID-19 pandemic, and the effect faded over time. PR re-incentivized PB only after the pandemic continued to deteriorate. For college students, emergency management measures taken by universities, such as campus lockdown and activity area restrictions, can re-trigger fluctuations in college students' PR, causing some abnormal behaviors among them (22, 23). Therefore, further discussion of the relationship between PR and PB is necessary during the COVID-19 pandemic's virus variant phase. Thus, hypothesis 1 was proposed:

- H1: Perceived risk is positively associated with preventive behavior.

The phenomenon that people's affective states change with a crisis is also included in the research category of the PMT (24, 25). According to Smith and Marinovich (26, 27), individuals tend to experience affective fluctuation in the face of crisis events. Those affects can be collectively referred to as positive affects (PA) and negative affects (NA) (28). PA refers to a kind of pleasant experience for people, including interest and gratitude (29). NA is usually undesirable emotions experienced after one fails to complete a task or a goal, such as fear, guilt, and anxiety (30). A study about the influence of environment risk on the public in Germany showed that the public's PR is closely linked with affects, which is that people's NA often accompanies PR (31). It is that NA often accompanies PR (32). And this consequence has been verified by a survey of the relationship between healthcare professionals' affects and their PR conducted during the pandemic. Healthcare professionals had a close and frequent contact with patients infected with COVID-19 pandemic, so they had a higher level of PR and depression, such as anxiety and stress (33). Another survey showed that, in terms of the COVID-19 pandemic, for each unit increase in PR, adults were twice ass likely to have depressive symptoms (34). Furthermore, PA and NA are independent and interrelated, and both of them fluctuate when people are in a public crisis (35). Jing (36) found that positive emotions were the most prominent online emotional characteristics of college students during college blockades. Kim (37) confirmed that, when college students faced PR of H1N1 influenza, PA (including interested, alert or curious) dominated. This view is also supported by communication research (38) that Twitter users noticed positive aspects of the situation; therefore, they expressed gratitude toward hospital staff and relief to their governments for their positive action in the epidemic. Those strong and determined affective states are important components of positive affects (39). These new findings about the relationship between PR and PA break stereotypes. The effect of the COVID-19 pandemic on NA (e.g., anxiety and worry) in college students has been supported by numerous empirical studies and experiences (40), while the effect of PR on PA in college students needs further investigation. Based on the above analysis, hypothesis 2 was proposed:

- H2: Perceived risk is positively associated with positive affect/negative affect.

The emergence of PA and NA triggers corresponding behaviors (41, 42). In major public events, when NA (e.g., worry and fear) emerges, people act accordingly for safety (43, 44). For example, a survey found that the public's willingness to wear masks during the COVID-19 pandemic was enhanced by the mediating effects of anger and anxiety (45). However, according to the theory of positive psychology, PA was strongly associated with effective coping behavior (46, 47), and PA was associated with an increase in PB (48). This indicates that PA promotes PB, which appears to be a paradox. To clarify the role of PA or NA in PB among college students, the following hypotheses were proposed:

- H3: Positive affect/negative affect is positively associated with preventive behavior.

- H4: Positive affect/negative affect plays a mediation role in the relationship between perceived risk and preventive behavior in college students.

The campus lockdown measures adopted by colleges and universities have brought many challenges to college students (49), such as irregular living habits, low learning efficiency, and difficulties in affect regulation (50), which has directly posed a great threat to the physical and mental health of college students. The benefits of physical exercise (PE) in improving the neuroendocrine system and the immune system, promoting mental health, and anti-anxiety have been proven (51). When college students had already perceived the risk of COVID-19, individuals with higher levels of physical exercise had lower negative affects (52). We can derive the conclulsion that physical exercise can partially ease the NA of college students (53). Meanwhile, some studies proved that healthy behavior could lead to preventive behavior (54). Healthy behavior, including physical activities, sleep, and diet, is the general name of various activities for people to strengthen the body system and maintain physical and mental health (55). As one of the most positive forms of physical activity (56), physical exercise can not only promote individuals' physical (57) and mental health (58) but also makes people realize the important role of healthy behavior (59, 60) during the pandemic. Some colleges and universities took health courses and sports activities as auxiliary means as adjust means to adjust the physical and mental state of college students during campus lockdown (61, 62) so as help them pursue a healthy life and promote the occurrence of preventive behaviors. Consequently, it is reasonable to assume that physical exercise contributes to PB among college students. Based on the above analysis, hypothesis 5 was proposed:

- H5: Physical exercise might moderate the mediation effect of positive affect/negative affect between perceived risk and preventive behavior of college students.

**Figure 1:**
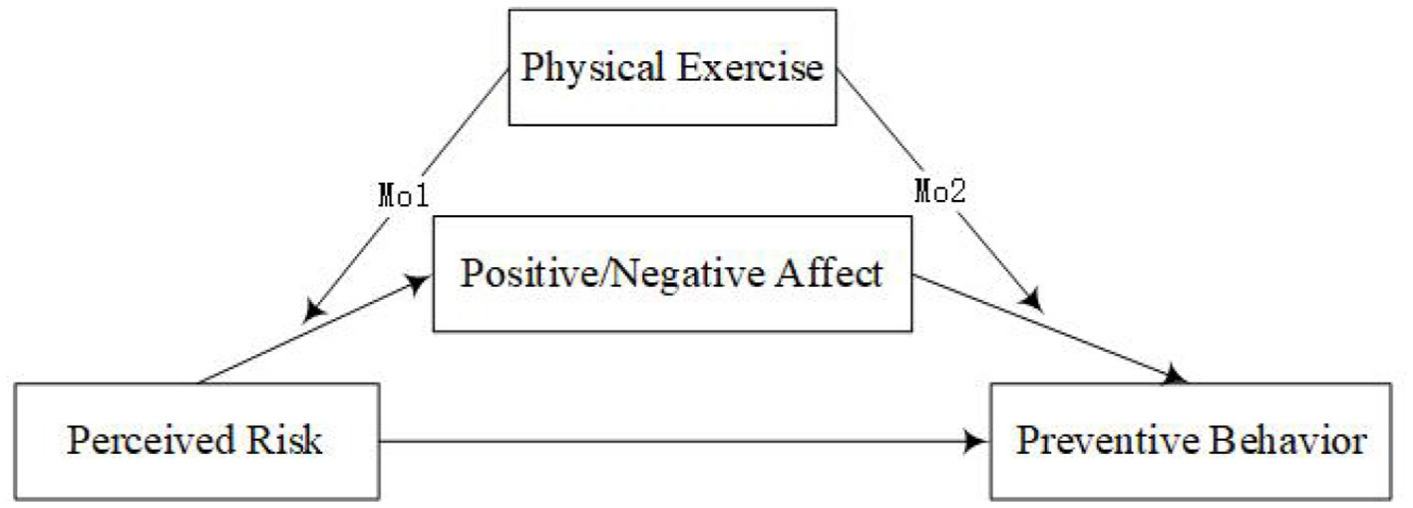
Conceptual framework of the proposed moderated mediation model. Mo1 refers to the first half of the mediating effect of physical exercise moderation; Mo2 refers to the second half of the mediating effect of physical exercise moderation.

Based on the earlier analysis, this study discusses how PR affects college students' PB, the mediation effect of PA/NA, and the moderating effect of PE (see Figure 1). The purpose of this study is to further reveal the internal relationship between PR and PB of college students during the pandemic and provide theoretical support and practical guidance for university managers from the perspective of psychological mechanisms.

## 2. Methods

### 2.1. Data collection and procedures

A stratified convenient sampling method was used (63), and the sample was limited to college students who were isolated on campus within Wuhan. The types of schools where the sample was located included 13 categories, such as comprehensive universities, science and technology universities, normal universities, medical universities, and finance and economics universities. Only one school in each category and 80–100 students from each school were selected for investigation. For this study, the questionnaires were sent to college students by virtue of an online survey platform (Survey Star, www.wjx.com). All participants were at least 18 years old, with Chinese as their native language, and could understand the items in the questionnaire. Since the time deviation of data collection may affect the correlation between variables and reduce the accuracy of conclusions, the survey time range was from 10 May 2022 to 10 June 2022, during which all universities were in lockdown. The interviewees were asked to recall the status of the previous week and fill in the questionnaire. Questionnaires were issued anonymously, and the content of the questionnaires can be used only for academic research. Finally, 1,144 questionnaires were collected in total, among which 25 were invalid (including 16 questionnaires with the same answers and nine questionnaires with response times of <30 s), and 1,119 were valid, with an effective recovery rate of 97.8%. The information on sociodemographic characterization, including gender, age, lockdown duration, the situation of a school lockdown, and major types, is summarized in Table 1.

**Table 1:** Sample descriptive statistics information (*N* = 1, 119).

**Variable**	***N* (%)**	**M ± SD**	**Min**	**Max**
**Gender**	1, 119 (100.0)	0.470 ± 0.499	0	1
Women	591 (52.8)			
Men	528 (47.2)			
**Age**	1, 119 (100.0)	22.04 ± 3.020	17	35
**Lockdown duration**	1, 119 (100.0)	3.260 ± 1.100	1	5
Less than 1 week	62 (5.5)	0.055 ± 0.229	0	1
2-3 weeks	216 (19.3)	0.193 ± 0.395	0	1
Approximately 1 month	371 (33.2)	0.332 ± 0.471	0	1
1–2 months	304 (27.2)	0.272 ± 0.445	0	1
More than 2 months	166 (14.8)	0.148 ± 0.356	0	1
**University lockdown situation**	1, 119 (100.0)	2.050 ± 0.730	1	3
Under strict lockdown	272 (24.3)	0.243 ± 0.429	0	1
Lockdown regularly	520 (46.5)	0.465 ± 0.499	0	1
Periodical Lockdown	327 (29.2)	0.292 ± 0.455	0	1
**Professional types**	1, 119 (100.0)	4.173 ± 1.209	1	5
The natural sciences	72 (6.4)	0.064 ± 0.245	0	1
Agricultural science	74 (6.6)	0.066 ± 0.249	0	1
Medical science	83 (7.4)	0.074 ± 0.262	0	1
Engineering and Technology Science	250 (22.3)	0.223 ± 0.417	0	1
Humanities and Social Sciences	640 (57.2)	0.572 ± 0.495	0	1
**Independent variable**				
Perceived risk	1, 119 (100.0)	2.722 ± 0.862	1	5.11
**Mediator variable**				
Negative affect	1, 119 (100.0)	2.970 ± 0.961	1	5
Positive affect	1, 119 (100.0)	2.856 ± 0.987	1	5
**Moderator variable**				
Physical exercise	1, 119 (100.0)	1.908 ± 0.922	1	3
**Dependent variable**				
Preventive behavior	1, 119 (100.0)	4.187 ± 1.615	1	7

N stands for the sample size; M stands for mean (the average value); SD stands for standard deviation; Min stands for minimum; Max stands for maximum.

SPSS 26.0 was adopted for descriptive statistical analysis and correlation analysis. Hierarchical linear regression analyses were conducted to further investigate the relationship between PR and PB, where the dependent variables in models 1–3, 4–5, and 6–7 were PB, PA, and NA, respectively. SPSS PROCESS 4.0 was used to test for the mediating effect of NA and PA, the moderating effect, and the moderated mediation effect of PE (64). Model 58 in the SPSS PROCESS guide was used to include both PAs and NAs in the model.

### 2.2. Instrumentation

#### 2.2.1. Perceived risk of COVID-19 pandemic scale

The PRCPS proposed by Xi et al. (65) was used to assess college students' PR during the campus lockdown. The scale was designed based on the Cognitive-Experiential Self-Theory (CEST) (66) and the Common-sense Model (CSM). It consists of nine items. According to Napper et al. (67) experience, the score assigned to each item is different. For example, *I think I am __ to have COVID-19* (1 = highly unlikely, 5 = very likely), *I am __ worried about COVID-19* (1 = never, 5 = always), *I find it is __ to imagine myself get COVID-19*. (1 = difficult, 4 = easy), *I'm sure I won't get COVID-19* (1 = totally disagree, 6 = totally agree), *I __ assume that I have COVID-19* (1 = never, 4 = often), *I feel vulnerable to COVID-19* (1 = totally disagree, 6 = totally agree), *No matter how small the chance is, I could get COVID-19* (1 = totally disagree, 6 = totally agree), *The possibility of myself getting COVID-19 is: ___* (1 = impossible, 6 = very likely), and *I've thought about that I have COVID-19* (1 = never, 4 = frequently). Before the scale was scored, the reverse item was corrected in advance. After summing up the scores of each item, the higher the total score is, the higher PR will be. Cronbach's α for this instrument was 0.834 (21).

#### 2.2.2. Positive and negative affect scale

The PANAS proposed by Watson et al. (68) was used to assess PA and NA. Huang et al. (69) revised and verified the applicability of the scale in the Chinese population (69). PA includes 10 descriptors such as interested, excited, and proud, and NA includes 10 descriptors such as disturbed, hostile, and ashamed. The respondent chose the corresponding answer according to the options (1 = totally disagree, 5 = totally agree); PA and NA were all tested by CFA, Cronbach's α for PA was 0.85 and 0.83 for NA (69).

#### 2.2.3. Preventive behavior scale

The PB was measured by PBS (21). This scale was proposed based on the recommended behavior of COVID-19 formulated by WHO (1). The PBS was used in a longitudinal survey of public PB in different stages of the COVID-19 pandemic in China and the United States (21), which included keeping social distance, avoiding crowds, wearing masks when going out, and washing hands frequently. Participants described the extent of their actions according to their situation in the last week (1 = never, 7 = extremely). Cronbach's α for this instrumentation was 0.837 (21).

#### 2.2.4. Physical activity rating scale

The PARS-3 (70) was used to assess participants' physical activity. Deqing (70) revised it and translated it into Chinese (70). Some Chinese scholars noticed its practical value and adopted it to measure the PE level of college students (71, 72). Thus, this scale was adopted to measure the PE level of college students in terms of intensity, duration, and frequency of PE. The respondents need to answer the following questions: “*How hard do you exercise?*”, “*How many minutes do you spend on the above intensity sports activities?*”, and “*How many times do you do the above sports activities in a month?*” The intensity of exercise was described as *lightly, low-intensity, moderate intensity, intensive and enduring, high-intensity but transitory*, and *high-intensity and enduring*. The duration of PE was described as ≤10, 11–20, 21–30, 31–59, and ≥ 60 min. The frequency of PE was described as *less than once a month, 2–3 times a month, 1–2 times a week, 3–5 times a week*, and *once a day*.

A 5-point Likert was used for scoring, and the total score was calculated according to the formula: Intensity × (Duration-1) × Frequency. The evaluation criteria for mild, moderate, and severe levels of PE were assessed as a total score of ≤19, 20–42, and ≥43, respectively. Cronbach's α for this instrumentation was 0.82 (70).

## 3. Results

### 3.1. Descriptive analysis and correlation analysis

Table 1 shows the demographic characteristics of the survey sample. Of the total participants, 47.2% of them were men and 52.8% were women, with an average age of 22.04. The lockdown durations of schools were <1 week (5.5%), 2–3 weeks (19.3%), approximately 1 month (33.2%), 1–2 months (27.2%), and more than 2 months (14.8%). In particular, 46.5% of the students reported that their schools were under regular lockdown and that students could enter and exit the school normally with the health code; 29.2% of the students reported that their schools were under periodical lockdown, and once there were new locally COVID-19 cases, the lockdown would be adopted until no new cases were detected; 24.3% of the students reported that their schools followed a regular and strict lockdown and prohibited access for visitation without a valid reason. The majority of the surveyed students belonged to Engineering and Technical Sciences (22.3%) and Humanities and Social Sciences (57.2%) disciplines.

Tables 1, 2 show the correlation coefficient and mean and standard deviations of each variable, respectively. According to Pearson's correlation results, the correlation coefficients among the variables ranged from 0.23 to 0.52, indicating a weak correlation between the variables. After analyzing the correlation results for the independent variable PR, the dependent variable PB, the mediating variable PA and NA, and the moderating variable PE, it was found that PR was positively correlated with PA, PE, and PB and negatively correlated with NA; PA was positively correlated with PE and PB, and negatively correlated with NA; NA was negatively correlated with PE and PB; PE was positively correlated with PB.

**Table 2:** Descriptive analysis and correlation analysis.

	**α^a^**	**CR**	**AVE**	**PR**	**NA**	**PA**	**PE**	**PB**	**KMO**	**χ^2^(df)**
PR	0.830	0.890	0.572	0.756^b^					0.960	23, 707.417(630)^***^
NA	0.920	0.921	0.539	0.317^***^	0.734^b^					
PA	0.940	0.937	0.597	−0.369^***^	−0.225^***^	0.773^b^				
PE	0.840	0.835	0.629	0.225^***^	−0.273^***^	0.398^***^	0.793^b^			
PB	0.840	0.804	0.507	0.307^***^	−0.370^***^	0.496^***^	0.517^***^	0.712^b^		

^***^p <0.001 (Pearson correlation). ^a^Cronbach's alpha coefficient. ^b^The square root of AVE. Independent variable: PR, perceived risk. Mediator variable: NA, negative affect; PA, positive affect. Moderator variable: PE, physical exercise. Dependent variable: PB, preventive behavior. CR, composite reliability; AVE, average variance extracted.

### 3.2. Regression analysis

To verify the direct effects among the variables, seven regression models were constructed with gender, age, and lockdown duration as control variables and PB, NA, and PA as dependent variables, respectively (see Table 3). In addition, the variance inflation factor (VIF) of the models revealed that the VIFs all ranged from 1.000 to 1.245, which are <2, indicating that there were no serious co-linear problems among the variables (73).

**Table 3:** Hierarchical linear regression analysis.

**Variable**	**Preventive behavior**			**Negative affect**		**Positive affect**	
	**Model 1**	**Model 2**	**Model 3**	**Model 4**	**Model 5**	**Model 6**	**Model 7**
Gender	−0.027	−0.022	−0.008	0.022	0.017	−0.028	−0.023
	[−0.171, 0.064]	[−1.005, 0.439]	[−0.140, 0.073]	[−0.074, 0.161]	[−0.077, 0.145]	[−0.175, 0.061]	[−0.155, 0.063]
Age	−0.011	−0.011	−0.004	−0.020	−0.019	−0.028	−0.029
	[−0.121, 0.083]	[−0.754, 0.499]	[−0.122, 0.063]	[−0.136, 0.068]	[−0.130, 0.063]	[−0.15, 0.054]	[−0.145, 0.045]
Lockdown duration	0.059^*^	0.072^*^	0.049^*^	−0.055	−0.068^*^	−0.001	0.014
	[0.001, 0.107]	[0.093, 0.749]	[−0.002, 0.095]	[−0.104, 0.003]	[−0.113, −0.011]	[−0.055, 0.052]	[−0.037, 0.062]
PR		0.309^***^	0.078^**^		−0.320^***^		0.370^***^
		[0.211, 0.304]	[0.157, 0.27]		[−0.375, −0.264]		[0.315, 0.424]
NA			−0.250^***^				
			[−0.355, −0.243]				
PA			0.411^***^ [0.234, 0.303]				
*R* ^2^	0.004	0.100	0.323	0.004	0.106	0.002	0.138
Δ*R*^2^	0.004	0.095	0.223	0.004	0.102	0.002	0.136
F	1.578	30.839^***^	88.327^***^	1.436	32.970^***^	0.580	44.523^***^
ΔF	1.578	118.126^***^	183.135^***^	1.436	127.085^***^	0.580	176.079^***^

^*^p <0.05; ^**^p <0.01; ^***^p <0.001. [, ] is the 95% confidence interval (95% CI).

PR, perceived risk; NA, negative affect; PA, positive affect; PB, preventive behavior. R^2^(R-squared) indicates the percentage of the variance of the dependent variable explained by the independent variable; ΔR^2^ (adjust R-squared) is used to evaluate the independent variable's contribution to the dependent variable degree of explanation. ΔF refers to the amount of change in F and represents the significance of the R^2^ difference.

PB was used as the dependent variable. Model 1 examined the effects of the control variables gender, age, and lockdown duration on PB, indicating that the effects of gender, age, and lockdown duration on PB were not significant (*F* = 1.578, *p* > 0.05). Based on model 1, model 2 took PR into consideration, indicating a significant positive effect of PR on college students' PB (β = 0.309, *p* < 0.001). Hypothesis 1 is confirmed.

Based on model 2, model 3 took the mediating variable PA and NA into consideration, indicating that NA has a significant negative effect on college students' PB (β = −0.250, *p* < 0.001) and PA has a significant positive effect on college students' PB (β = 0.411, *p* < 0.001). It is invalid that the NA positively affects PB in hypothesis 3. Thus, hypothesis 3 is partially confirmed.

NA was used as the dependent variable. Model 4 showed that gender, age, and lockdown duration have no significant effect on college students' NA (*F* = 1.436, *p* > 0.05). Based on model 4, model 5 took PR into consideration, indicating that PR has a significant negative effect on the NA of college students (β = −0.320, *p* < 0.001). It is invalid that the PR positively affects NA in hypothesis 2. PA was used as the dependent variable. Model 6 indicated that gender, age, and lockdown duration have no significant effect on PA among college students (*F* = 0.580, *p* > 0.05). The addition of PR indicates that PR has a significant positive effect on PA among college students (β = 0.370, *p* < 0.001). Hypothesis 2 is partially confirmed.

### 3.3. Moderated mediation analysis

Table 4 shows the results of the mediation effect of PA and NA on PR and PB among college students. The results show that the association between PR and PB *via* the mediator (NA) is 0.080, with a confidence interval of [0.061, 0.102], excluding 0. It indicates that NA plays a significant role in the association between PR and PB, and this indirect effect accounts for 26.2% of the total effect. The association between PR and PB *via* the mediator (PA) is 0.152, with a confidence interval of [0.124, 0.180], excluding 0. It indicates that PA also plays a significant role in the association between PR and PB, and this indirect effect accounts for 49.5% of the total effect. Hypothesis 4 is confirmed. The confidence interval for the difference in the association between PR and PB *via* the mediator (NA and PA) is [−0.106, −0.037], indicating that the mediation effect of PA is significantly higher than that of NA.

**Table 4:** Mediating effect test.

	**Indirect effect**	**SE**	**95% CI**	**Relative effect**
Total indirect effect	0.232	0.018	[0.199, 0.267]	75.70%
NA	0.080	0.010	[0.061, 0.102]	26.20%
PA	0.152	0.015	[0.124, 0.180]	49.50%
(C1)	−0.072	0.018	[−0.106, −0.037]	
Total effect	0.308			

(C1): NA minus PA. SE, standard error; CI, confidence interval. Relative effect = |total indirect effect| / |total effect| ^*^ 100%. Mediators variable: NA, negative affect; PA, positive affect. Dependent variable: PB, preventive behavior.

Table 5 shows the results of the moderating effect of PE. In model 8, the interaction terms of NA and PE have a significant negative effect on PB (β = −0.094, *t* = −3.779, *p* < 0.001), indicating that PE has a significant negative moderating effect between NA and PB; the interaction terms of PA and PE has a significant positive effect on PB (β = 0.075, *t* = 2.770, *p* < 0.01), indicating that PE has a significant positive moderating effect between PA and PB. In model 9, the interaction terms of PR and PE has a significant negative effect on NA (β = −0.162, *t* = −5.634, *p* < 0.001), indicating that PE has a significant negative moderating effect between PR and NA. In model 10, the interaction terms of PR and PE has a significant positive effect on PA (β = 0.163, *t* = 6.018, *p* < 0.001), indicating that PE has a significant positive moderating effect between PR and PA.

**Table 5:** Standardized coefficients for the moderated mediation model.

**Variable**	**Coeff**	**SE**	***t***	**95% CI**
**Model 8** ^a^				
PR	0.036	0.026	1.393	[−0.015, 0.087]
NA	−0.170	0.026	−6.68^***^	[−0.22, −0.120]
PA	0.271	0.027	10.046^***^	[0.218, 0.323]
PE	0.353	0.026	13.704^***^	[0.302, 0.403]
Int_1	−0.094	0.025	−3.779^***^	[−0.142, −0.045]
Int_2	0.075	0.027	2.770^**^	[0.022, 0.127]
constant	−0.055	0.026	−2.143^*^	[−0.106, −0.005]
R^2^	0.422			
F	135.513^***^			
**Model 9** ^b^				
PR	−0.227	0.029	−7.802^***^	[−0.284, −0.170]
PE	−0.219	0.028	−7.798^***^	[−0.274, −0.164]
Int_3	−0.162	0.028	−5.634^***^	[−0.219, −0.106]
constant	0.037	0.028	1.299	[−0.002, 0.092]
R^2^	0.167			
F	74.589^***^			
**Model 10** ^c^				
PR	0.252	0.027	9.220^***^	[0.198, 0.305]
PE	0.338	0.026	12.807^***^	[0.286, 0.390]
Int_3	0.163	0.026	6.018^***^	[0.110, 0.216]
constant	−0.037	0.026	−1.388	[−0.088, 0.015]
R^2^	0.264			
*F*	133.584^***^			

^*^p <0.05; ^**^p <0.01; ^***^p <0.001. ^a^The dependent variable is PB. ^b^The dependent variable is NA. ^c^The dependent variable is PA. PR, perceived risk; NA, negative affect; PA, positive affect. Moderator variable: PE, physical exercise. Int_1, NA^*^PE; Int_2, PA^*^PE; Int_3, PR^*^PE. Coeff stands for coefficient. SE, standrad error; CI, confidence interval. R^2^ indicates the percentage of the variance of the dependent variable explained by the independent variable.

**Figure 2:**
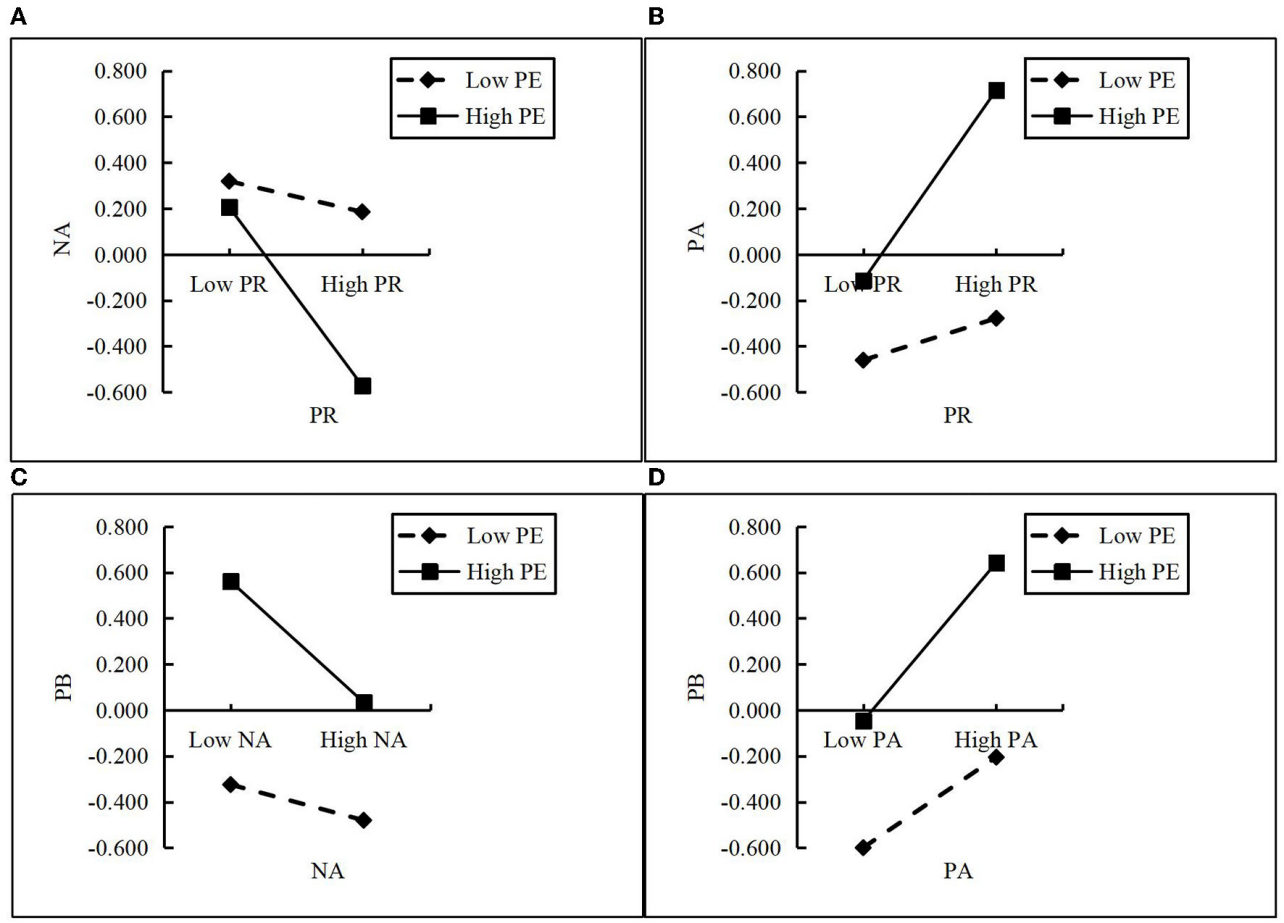
Simple slope indicating the moderation effects. **(A)** Moderating effect of physical exercise on perceived risk and negative affect; **(B)** Moderating effect of physical exercise on perceived risk and positive affect; **(C)** Moderating effect of physical exercise on negative affect and preventive behavior; **(D)** Moderating effect of physical exercise on positive affect and preventive behavior. PR, perceived risk; NA, negative affect; PA, positive affect; PE, physical exercise; PB, preventive behavior.

Figure 2 shows a simple slope chart of the moderating effects. The results show that, under the effect of high-intensity PE, PR has a stronger negative effect on NA and a stronger positive effect on PA, while NA has a stronger negative effect on PB, and PA has a stronger positive effect on PB.

A bootstrap method was used to further confirm the presence of mediating effects whose low and high values were calculated based on the mean ± 1 standard deviation. The results of the mediated moderation effect are demonstrated in Table 6. When levels of physical exercise are low (M – 1SD), the mediating effect of NA between PR and PB is not significant (β = 0.005, 95% CI = [−0.002, 0.014]). When levels of physical exercise are high (M + 1SD), the mediating effect of NA between PR and PB is significant (β = 0.103, 95% CI = [0.075, 0.134]). The difference value of the mediated effect of NA is significant at different levels of physical exercise (Δβ = 0.097, 95% CI = [0.070, 0.128]). Thus, the mediating effect of NA between PR and PB is moderated by physical exercise. When levels of physical exercise are low (M – 1SD), the mediating effect of PA between PR and PB is significant (β = 0.018, 95% CI = [0.003, 0.034]). When levels of physical exercise are high (M + 1SD), the mediating effect of PA between PR and PB is significant (β = 0.143, 95% CI = [0.113, 0.179]). The difference value of the mediating effect of PA is significant at different levels of physical exercise (Δβ = 0.125, 95% CI = [0.090, 0.165]). The mediating effect of PA between PR and PB is moderated by physical exercise. Thus, hypothesis 5 is confirmed.

**Table 6:** Mediated moderation effect results in analysis.

**Path**	**PE**	**Effect**	**SE**	**95% CI**
PR → NA → PB	PE (M−1SD)	0.005	0.004	[−0.002, 0.014]
	PE (M+1SD)	0.103	0.015	[0.075, 0.134]
	(C1)	0.097	0.015	[0.070, 0.128]
PR → PA → PB	PE (M−1SD)	0.018	0.008	[0.003, 0.034]
	PE (M+1SD)	0.143	0.017	[0.113, 0.179]
	(C1)	0.125	0.019	[0.090, 0.165]

(C1): PE (M+1SD) minus PE (M-1SD). SE, standrad error; CI, confidence interval.

## 4. Discussion

Based on the PMT, this study constructed a moderated mediation model with PA and NA as the mediating variables and with physical exercise as moderating variable, and the influence of college students' PR on PB was discussed.

Our results support the notion that PR is positively associated with PB (74). College students with higher PR scores would adopt more active PB (e.g., wearing masks and paying attention to personal hygiene) to keep themselves safe from the threat of infectious diseases, which is consistent with studies conducted in the earlier stage of COVID-19 (75). The results also support the protection motivation theory (PMT) in some aspects; that is, once a certain threshold of PR in events is exceeded, countermeasures will be adopted to reduce or avert the risk. During the survey, new cases of COVID-19 consecutively emerged in Wuhan city (76, 77), and college students believed that they were more vulnerable to infectious diseases than before (11, 21). Although the epidemic lasted 2 years, when school administrators took strict management measures against COVID-19, students' PR levels improved and PB also appeared.

The results could indicate that higher levels of PR cause lower NA and higher PA. The relationship between PR and NA in this study is partly different from the previous studies (78, 79), which might be explained by different investigated objects. Across the globe, studies confirmed that people's NA erupt and PA rarely appear under the condition of a high level of PR related to COVID-19, whether it is children (80), older people (81), or young people (82). However, this study is special in this respect. College students in Wuhan were one of the first groups to experience the serious influence of the pandemic, and they could prove the effectiveness of closed-off management in suppressing the continued spread of the pandemic, which gave them more reason to support campus lockdown than the general public (83, 84). In addition, Eichenberg (78) found that economic worries are also one of the main reasons for negative emotions. At the same time, college students suffered less economic loss during COVID-19 (85), which can be considered the main reason that PR is positively associated with PA and is negatively associated with NA.

The results could indicate that college students' PA was positively associated with PB and NA was negatively associated with PB. This is consistent with the proposition of positive psychology (46, 47) that PA can pre-process individual cognition and behavior to improve the ability to cope with emergency events (86). PA might strengthen college students' confidence in coping with stressful events, help them adapt to the campus lockdown, and become more aware of behavioral control. College students with high PA can deal well with the rapid changes in the external environment with a stable attitude (69) and exhibit more positive PBs (67).

The results about mediating effect showed that PA and NA partially mediated the relationship between PR and PB among college students, and the value of the indirect effect of PA is greater than that of NA. This finding provides more evidence for emerging research. It reveals the differential influences of PA/NA on PB related to PR (21). That is, college students' PR might increase PB by reducing NA and increasing PA. NA strengthens counterproductive work behavior (87), like violating PB in the COVID-19 pandemic, and meanwhile negatively affects health behavior (88). Individuals with higher levels of PA are more likely to care for themselves (89) and show great potential in health behavior learning (90). PA can help people cope with PR, especially after the COVID-19 lockdown (91). In this study, the influence of PA on PB was greater than that of NA. The researchers believed that this may be because PA was an important source of people's spiritual strength, which can pre-process individual cognition, emotions, and behaviors to establish and expand personal and social resources (92). People with high PA could have more psychological preparation and health behavioral response than those with NA when dealing with emergencies (93).

The results of the moderating effect showed that PE moderated not only the first half of the mediated effect pathway but also the second half of the mediated effect pathway. Specifically, the mediating effect of PA and NA between PR and PB was significantly enhanced in college students with high levels of PE. This may be because people who participate more in physical exercise may be more inclined to pursue a healthy lifestyle, are more likely to perceive risks, feel more able to adhere to preventive behavior, have a more positive impact, and actively participate in preventive behavior. Some facts proved that, through higher levels of risk information perceived from the COVID-19 pandemic, and college students might be able to generate higher levels of PAs and lower levels of NAs with the help of PE during the campus lockdown, which then led to active PBs. This confirmed the role of PE on mental health (51). PE can help college students enhance their physical health (94), release NA, gain interpersonal interaction, build better relationships with peers, and obtain much social support (95). College students who participate in PE regularly can better regulate their bad mood and adopt a more active PB to cope with the risk of the COVID-19 pandemic. Extending the opening hours of sports venues on campus, conducting online sports guidance (96), holding online sports competitions, such as online marathons (all participants need to upload real-time mileage within the specified time (97), and holding sports exchange forums on campus (98, 99) are considered to be effective measures to encourage college students to take more physical exercises.

## 5. Conclusion

In the context of ongoing campus lockdown due to the COVID-19 pandemic, this study mainly determined the relationship between college students' PR and PB, revealed the potential mediating role of PA and NA on the relationship between PR and PB among college students, and discussed the potential moderating role of PE in the aforementioned mediating role. We built a model which is composed of five hypotheses and partly verified them with 1,119 valid responses. The results showed that PR is not only directly and significantly associated with PB but also influences PB through PA and NA, and the effect of PA is greater than that of NA. Furthermore, PE moderates the mediating role of PA and NA between PR and PB in college students. These findings contribute to illuminating how PA/NA mediates college student's PR and PB and how PE participates in the relationship mentioned earlier.

College students' PR is positively associated with PB, but measures to increase college students' PR should not be limited to adopting campus lockdowns in all respects. The experience of Wuhan provides an excellent example of public crisis events, and adopting more flexible measures in lockdown is effective for enduring the existence of the COVID-19 pandemic; the important intermediary mechanism of PA/NA in this study requires college administrators to take the obligation to help college students establish a correct concept of affects management and provide a convenient channel for help on this basis of preventing them from missing the opportunity of psychological intervention when encountering emergent public crisis. The effect of PE is confirmed in this study. Based on the important function of PE on PR and PB, college administrators should provide more sports venues and prolong the timing of availablity, and hold sports games of different scales to encourage college students to take more PE to give full play the role of PE and enhance college students' PB in the COVID-19 pandemic.

## 6. Limitation and prospects

First, the utilization of cross-section data in this article cannot guarantee the evaluation of causal relationships among variables, which would inevitably result in inaccurate research conclusions. Future research is suggested to adopt a longitudinal research method which is necessary to substantiate the findings from the present study and further improve the validity of the conclusion. Second, in terms of the respondents, college students in Wuhan are a typical representative group on the topic of COVID-19; thus, this study is not generalizable to other populations than students. Third, due to the limitation in the range of variable selection, social management factors such as public opinion and government authority are excluded, which can be further studied.

## Data availability statement

The raw data supporting the conclusions of this article will be made available by the authors, without undue reservation.

## Ethics statement

Ethical review and approval was not required for the study of human participants in accordance with the local legislation and institutional requirements. The participants provided their written informed consent to participate in this study.

## Author contributions

LZ: conceptualization, manuscript writing, and revision. XC: data analysis, revision, and proofreading. ZL: translation and proofreading. All the authors participated in the article and approved the submitted version.
